# New challenge for the health care system in Iran: The need to prepare for the monkeypox virus

**DOI:** 10.3389/fmed.2022.974169

**Published:** 2022-09-26

**Authors:** Mohammad Ali Zakeri, Abbas Zakeri Bazmandeh, Mahmood Kahnooji, Mahlagha Dehghan

**Affiliations:** ^1^Social Determinants of Health Research Center, Rafsanjan University of Medical Sciences, Rafsanjan, Iran; ^2^Non-communicable Diseases Research Center, Rafsanjan University of Medical Sciences, Rafsanjan, Iran; ^3^Department of Medical Nanotechnology, School of Advanced Medical Sciences and Technologies, Shiraz University of Medical Sciences, Shiraz, Iran; ^4^Department of Internal Medicine, Faculty of Medicine, Rafsanjan University of Medical Sciences, Rafsanjan, Iran; ^5^Nursing Research Center, Kerman University of Medical Sciences, Kerman, Iran

**Keywords:** care, challenge, health, monkeypox, virus

## Introduction

Human monkeypox was first reported as a common human-animal disease in 1970 in Central Africa (1). It is caused by an orthopox virus that can induce complications such as pneumonitis, encephalitis, sight-threatening keratitis, and secondary bacterial infections (1, 2). The published mortality rate varies considerably and is reported to vary between 0%−10% (3). Monkeypox can cause a severe clinical condition with systemic signs and symptoms. A study by Huhn et al. (2) revealed that 15% of monkeypox patients (34 patients) developed severe symptoms and 26% were hospitalized for more than 48 h. Patients under the age of 18 are more likely to be admitted to the intensive care unit (2).

## Subsections relevant for the subject

According to the WHO, on May 29, 2022, monkeypox was reported in 23 countries; on the whole, a total of 257 laboratory-confirmed cases and about 120 suspected cases have been reported. However, more cases are expected to be identified in countries that have not yet reported cases. Cases have been reported mainly among homosexual Men having Sex with Men (MSM) (4). A new study by Adler et al. found that infection prevention and control are difficult due to long-term upper respiratory tract viral DNA shedding after skin lesion resolution (5). However, of the three reported cases of acquired monkeypox in the UK, one was a health care worker who received the virus in a hospital setting (5). Social media in Iran deals with news related to monkeypox cases. A wave of questions has emerged among people about the effects and problems caused by this disease, which needs to be considered by the authorities to manage and prevent possible dangers. Besides, in the UAE, in the vicinity of Iran, three cases of monkeypox have been reported (6).

## Discussion

Some societies have not yet been able to fully eradicate the coronavirus epidemic, like Iran, which is still grappling with the COVID-19 pandemic. In the meanwhile, the presence of monkeypox disease and the difficulties it causes can raise significant concerns in those who are exposed to it. The review of the literature shows that with the occurrence of epidemics and critical conditions such as the epidemic of the COVID-19 disease, a wave of fear, anxiety (7), stress (8) and then many mental disorders are experienced in the communities (9) or treatment staff (10). Although we may not experience a large-scale epidemic like the COVID-19 disease of monkeypox, however, the COVID-19 epidemic taught us that we should be more prepared for unknown epidemics and take necessary precautions in any situation.

Although no case of monkeypox has been reported in Iran yet, there exists the possibility of the spread of the virus to Iran. This issue has sounded the alarm for the Iranian health care system that it may encounter the possibility of infection with monkeypox. Thus, it is necessary to pay more attention to the possible effects of the spread of this virus and how to deal with it. Monkeypox can impose several challenges for the Iranian healthcare system. The more important of these challenges are:

### Preparation of Iran's health care system

Iran's health care system and medical personnel are still involved in the COVID-19 pandemic, which itself requires attention and various preventive-therapeutic measures. Therefore, the additional involvement of this system in another disease increases the problems and workload of medical staff. So, to prevent the spread of monkeypox in Iran, healthcare managers, in addition to focusing on coronavirus disease, should make more efforts to organize and plan to control and manage a new critical situation called monkeypox.

### Community preparation

Some studies in recent years have demonstrated that with the apparent increase in monkeypox cases, there is the possibility of further spread of the disease; also, the level of concern about the spread of this emerging disease has increased (3). A giant strand can be taken to control and prevent this disease and possible complications such as fear, anxiety, and depression by increasing public knowledge and awareness about disease recognition and implementing safety protocols. The mass media can play an important role in guiding and organizing education and controlling potential crises in affected and non-affected individuals. Nonetheless, fear of social stigma associated with being diagnosed with monkeypox can cause anxiety or depression in afflicted individuals. It was documented in 2018 about patients with monkeypox that the need for psychiatric counseling was felt in more than a quarter of patients admitted with monkeypox (11). In the case of an incidence of monkeypox disease, the situation can become more difficult for society to deal with the disease due to the current unfavorable economic situation in Iran and the existence of various sanctions on Iran.

### Necessary provisions, prevention facilities, and equipping medical centers

The experience of facing the effects of the COVID-19 disease showed that epidemics can disrupt the work of the treatment system (12) and the needs of patients in terms of management, patient care, equipment supply, and human resources (13). This condition may also be seen in the face of monkeypox disease. Therefore, this idea has already been proposed for the SARS-CoV-2 coronavirus disease, which means it is necessary to urgently improve health systems during emergencies.

Many countries lack new experience and special knowledge about monkeypox disease to identify cases, treat patients, and prevent further spread of the monkeypox virus. Coping with the monkeypox virus requires planning, monitoring, and attempts to diagnose and control the infection. In addition, the epidemiology and ecology of the virus need to be considered in designing prevention programs (3).

### Preparing medical personnel

In the face of the monkeypox virus, healthcare professionals should take standard precautions, contact, and droplets. These precautions can be applied to any health center, including outpatient services and hospitals. Standard precaution measures include strict hand hygiene, proper handling of contaminated medical equipment, and cleaning and disinfection of environmental surfaces (4). Hence, it is necessary for health and care staff to have sufficient knowledge about the signs and symptoms of monkeypox disease and to assist in the timely diagnosis and quarantine of suspected patients. Some of the most important measures in hospitals to control and prevent monkeypox include proper monitoring systems; efforts to track the source of the virus in positive cases; equipped hospital isolation units; and inter-ward coordination.

### Crisis management

Most available data on monkeypox indicates incomplete information on endemic countries and single afflictions or recurrences that do not provide accurate information on monkeypox. There is a large gap in information on monkeypox and new epidemiological changes, clinical manifestations, and ways of transmitting the disease, which is itself a reason for further efforts to prepare for a sudden outbreak. Lessons learned from the COVID-19 epidemic emphasize the importance of new and comprehensive approaches to timely crisis management. Periods of severe political instability, increasing poverty, and environmental disturbances may lead to frequent human contact with host animals, resulting in increased Zoonosis diseases (diseases shared between animals and humans).

As a result, it is necessary to evaluate the etiological and ecological risk factors involved in human infections (14). Politicians need to have a written plan for virus screening, health care design, strategies for coping and controlling the disease, and follow up on problems associated with the disease. WHO urges immediate action, including the following: (1) providing accurate information to those most vulnerable to monkeypox; (2) preventing further spread among vulnerable populations; and (3) protecting health workers at the forefront of that action (4). Risks caused by the spread of monkeypox in Iran may impose several challenges for the health care system. Finally, governments and managers should focus more on being prepared to prevent and control monkeypox by anticipating and implementing the necessary measures.

## Author contributions

MZ, AZB, MD, and MK conceived the idea and designed the study. All authors contributed to the manuscript writing and approved the manuscript submission.

## Conflict of interest

The authors declare that the research was conducted in the absence of any commercial or financial relationships that could be construed as a potential conflict of interest.

## Publisher's note

All claims expressed in this article are solely those of the authors and do not necessarily represent those of their affiliated organizations, or those of the publisher, the editors and the reviewers. Any product that may be evaluated in this article, or claim that may be made by its manufacturer, is not guaranteed or endorsed by the publisher.
